# Negative correlation between soil salinity and soil organic carbon variability

**DOI:** 10.1073/pnas.2317332121

**Published:** 2024-04-26

**Authors:** Amirhossein Hassani, Pete Smith, Nima Shokri

**Affiliations:** ^a^The Climate and Environmental Research Institute NILU, Kjeller 2027, Norway; ^b^Institute of Biological and Environmental Sciences, School of Biological Sciences, University of Aberdeen, Aberdeen AB24 3UU, United Kingdom; ^c^Institute of Geo-Hydroinformatics, Hamburg University of Technology, 21073 Hamburg, Germany

**Keywords:** soil organic carbon, soil salinity, environmental impact, carbon cycle, biogeochemistry

## Abstract

Soil organic carbon (SOC) is vital for terrestrial ecosystems, affecting biogeochemical processes, and soil health. It is known that soil salinity impacts SOC content, yet the specific direction and magnitude of SOC variability in relation to soil salinity remain poorly understood. Analyzing 43,459 mineral soil samples (SOC < 150 g kg^−1^) collected across different land covers since 1992, we approximate a soil salinity increase from 1 to 5 dS m^−1^ in croplands would be associated with a decline in mineral soils SOC from 0.14 g kg^−1^ above the mean predicted SOC (${\overset{¯}{\mathrm{SOC}}}_{c}$ = 18.47 g kg^−1^) to 0.46 g kg^−1^ below ${\overset{¯}{\mathrm{SOC}}}_{c}$ (~−430%), while for noncroplands, such decline is sharper, from 0.96 above ${\overset{¯}{\mathrm{SOC}}}_{\mathrm{nc}}$ = 35.96 g kg^−1^ to 4.99 below ${\overset{¯}{\mathrm{SOC}}}_{\mathrm{nc}}$ (~−620%). Although salinity’s significance in explaining SOC variability is minor (<6%), we estimate a one SD increase in salinity of topsoil samples (0 to 7 cm) correlates with respective $\overset{¯}{\mathrm{SOC}}$ declines of ~4.4% and ~9.26%, relative to ${\overset{¯}{\mathrm{SOC}}}_{c}$ and ${\overset{¯}{\mathrm{SOC}}}_{\mathrm{nc}}$. The $\overset{¯}{\mathrm{SOC}}$ decline in croplands is greatest in vegetation/cropland mosaics while lands covered with evergreen needle-leaved trees are estimated with the highest $\overset{¯}{\mathrm{SOC}}$ decline in noncroplands. We identify soil nitrogen, land cover, and precipitation Seasonality Index as the most significant parameters in explaining the SOC’s variability. The findings provide insights into SOC dynamics under increased soil salinity, improving understanding of SOC stock responses to land degradation and climate warming.

## Significance

Soil organic carbon (SOC) is integral to terrestrial ecosystems, influencing soil health and ecological processes. Soil salinity, a growing concern due to climate change and human activities, can have either detrimental or beneficial effects on SOC. This analysis sheds light on the intricate relationship between salinity and SOC, revealing that soil salinity is negatively correlated with SOC content. Such insights are vital for addressing global challenges, including land degradation and climate change.

Soil organic carbon (SOC) is a crucial component of terrestrial ecosystems and plays a significant role in numerous biogeochemical processes (1). It constitutes a dynamic pool of carbon intricately linked to climate regulation, nutrient cycling, and soil health (2). Soil carbon is the main component of soil organic matter (SOM), which encompasses various organic materials (OM) derived from plant and animal residues, microbial biomass, and other decomposed organic substances (3, 4).

Soil salinity is a measure of the concentration of soluble salts in the soil solution (5, 6). Salinity can occur naturally in certain regions due to arid or semiarid conditions, leading to the accumulation of salts through processes like mineral weathering or anthropogenic activities like irrigation and improper land management (7). Excessive soil salinity is a significant environmental issue worldwide (8), negatively impacting soil fertility, plant growth, and overall ecosystem productivity (9–11).

Explaining the interplay between soil salinity and SOC content is essential for understanding the potential impacts of soil salinity on carbon sequestration, climate change mitigation efforts, and the stability of terrestrial carbon stocks (12). However, the relationship between soil salinity and SOC content, particularly in field conditions and at large geographical scales, is intricate and can exhibit variable effects (13, 14). The net effect of soil salinity on SOC content indeed remains complex which necessitates further research to gain a complete understanding (12, 15, 16).

High salinity levels can inhibit microbial activity, reducing the decomposition of OM in the soil (17–19). This slowdown in decomposition rates can result in the accumulation of SOC, as OMs persist for longer periods before being fully broken down (20). Elevated soil salinity can induce the flocculation of clay particles into aggregates, potentially limiting substrate availability and slowing down the decomposition of SOM (12). Moreover, soil salinity can promote the binding of organic carbon to soil particles through cation bridging at higher electrolyte concentrations. As a result of this enhanced carbon stabilization, saline-sodic soils demonstrate lower SOC loss than sodic soils (21). All these mechanisms are expected to lead to SOC accumulation/increase over time.

On the other hand, moderate to high salinity can negatively impact vegetation, reducing plant biomass, and root exudates (15, 22, 23). In highly saline environments, plant growth and productivity may be limited due to the osmotic stress caused by excess salts (24, 25). Fewer OMs and litter entering the soil from plants lead to a potential decline in the SOC content (26). Additionally, salinity-induced soil degradation, such as surface crusting, can increase soil erosion rates, leading to the loss of OM and reducing SOC (16, 27). Extreme salinity can lead to shifts in microbial communities (18). Some halophilic (salt-loving) microorganisms may become dominant, which could impact the decomposition of SOM differently than in nonsaline soils, potentially affecting the SOC content (18).

## The Significance of Soil Salinity in Predicting SOC

The net effect of soil salinity on the SOC content is thus a balance between the positive and negative influences. It depends on the specific interacting factors, including climate, soil type, vegetation, land use practices, and other soil physicochemical properties.

We analyzed a vast dataset comprising 43,459 soil samples from diverse biomes and land covers, particularly Europe, collected since 1992. The soil samples were collected from various depths and were obtained from well-established soil profile databases such as LUCAS (28) (*n* = 31,168) and WoSIS (29) (*n* = 12,291). These samples included soil salinity measurements, represented by soil–water extract electrical conductivity (EC) (dS m^−1^) and SOC content (g kg^−1^) at each sampling location. The primary objective of our research was to examine the correlation between soil salinity and SOC in mineral soils (SOC < 150 g kg^−1^) by controlling other environmental parameters that could explain the variability in SOC. Our assumption was that a steady-state balance exists between soil, land, and climate at the sampling locations over extended periods. However, this assumption may not necessarily be valid, especially in croplands, where over short time scales land and crop management practices may play a more significant role in explaining the variability in SOC. Accordingly, we separated the analysis into croplands (*n* = 25,634) and noncroplands (*n* = 17,825). The interpretation of the developed statistical analysis enabled us to identify additional significant environmental factors and their relationship with SOC content.

To account for the complex nature of soil systems, we considered several critical environmental factors that may interact with and are correlated with the behavior of OC in the soil including climate, vegetation cover, land use practices, soil texture, soil physio-chemical properties, drainage patterns, soil moisture levels, and land management strategies. We employed general additive models (GAMs) to investigate the relation between soil salinity and SOC content while considering potential nonlinear patterns and interactions with other parameters. Given the innate correlation between these variables at large scale and the possibility of concurvity, or fundamental associations between these variables, we first ranked covariates based on their minimum redundancy and maximum relevance to SOC using MRMR (Minimum Redundancy Maximum Relevance) algorithm (30). For two major land covers, croplands, and noncroplands, we fitted separate GAMs to the SOC of soil samples with SOC < 150 g kg^−^^1^, as the target variable.

In both cases, certain environmental variables were identified as the most influential in explaining SOC variability (*SI Appendix*, Fig. S1). Specifically, the covariates identified as significant in both croplands and noncroplands were soil total soil nitrogen (N) content (g kg^−1^), land cover, sample depth (cm), sand content (%) in the fine earth fraction (particles < 2 mm), soil pH (measured in aqueous solution), and precipitation Seasonality Index (SI)— representing the variation in precipitation throughout the year within an area. Soil salinity emerged as one of the primary covariates in both land types. Moreover, leaf area index (LAI) was found to be significant for croplands, while topographic hill slope emerged as influential for noncroplands. One result here is that the precipitation SI emerges as a more significant covariate compared to annual precipitation and long-term air temperature.

**Fig. 1.**
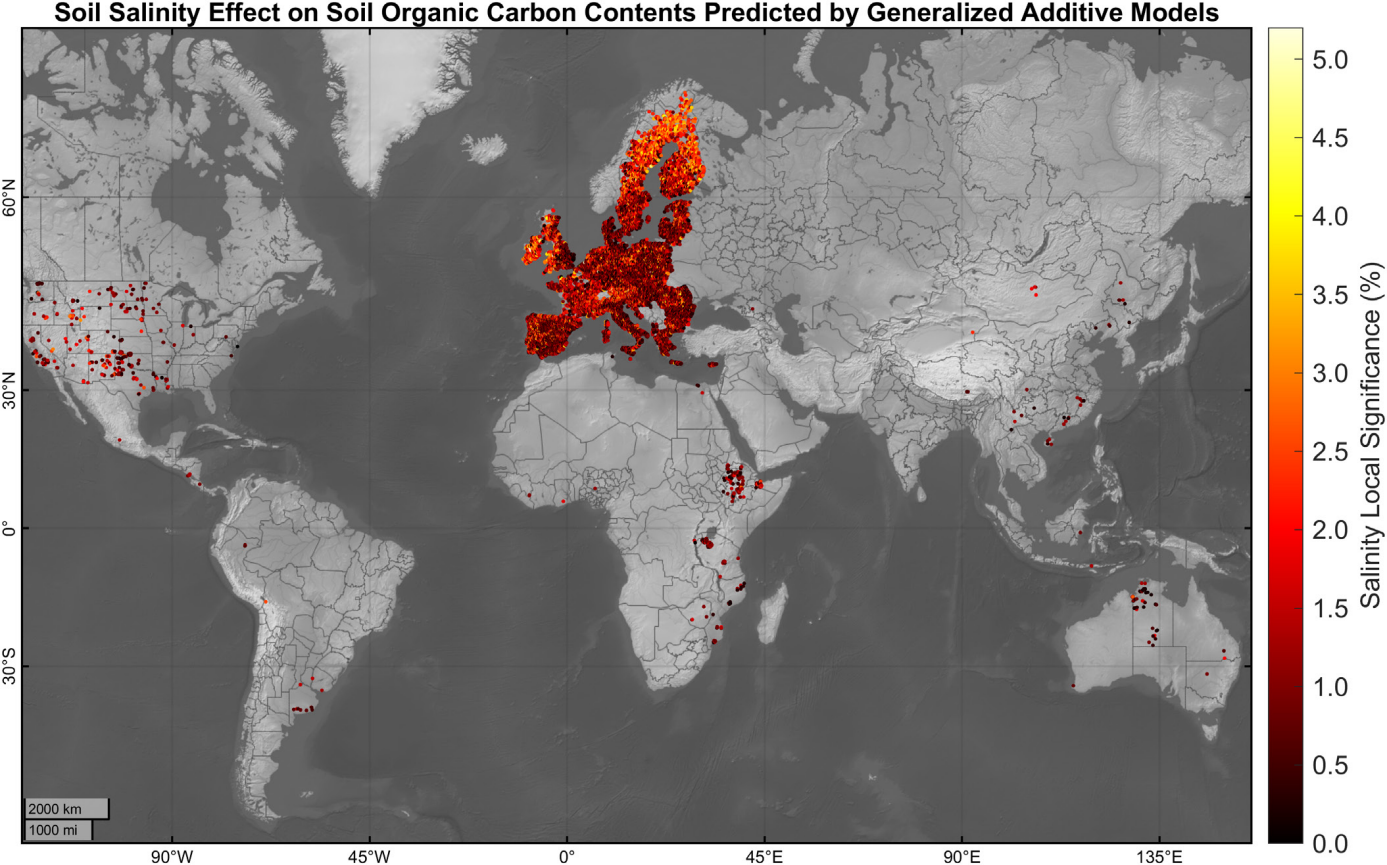
Vertically averaged local significance of soil salinity in prediction of SOC content using GAMs. Soil salinity is represented by EC of different soil–water extracts (dS m^−1^). For every observation, the percentage of local covariate significance for predicting SOC was calculated by dividing the absolute effect of that specific covariate by the sum of the absolute effects of all other covariates, excluding the intercept.

Using GAMs allowed us to discern the specific local significance of different variables, including soil salinity, in prediction of SOC contents at varying levels of soil–water extracts (Fig. 1). For every observation, the percentage of local covariate significance for predicting SOC was calculated by dividing the absolute effect of that specific covariate by the sum of the absolute effects of all other covariates, excluding the intercept. The results indicated that the local significance of soil salinity in prediction of SOC contents ranged from 0% to ~6% across different soil-water extracts. On average, the salinity’s mean significance in prediction of SOC content was ~1.13% (SD = ~0.94%). The low mean significance of salinity suggests that other factors, such as climate, land cover, or soil texture, may have more substantial effects in prediction of SOC contents.

**Fig. 2.**
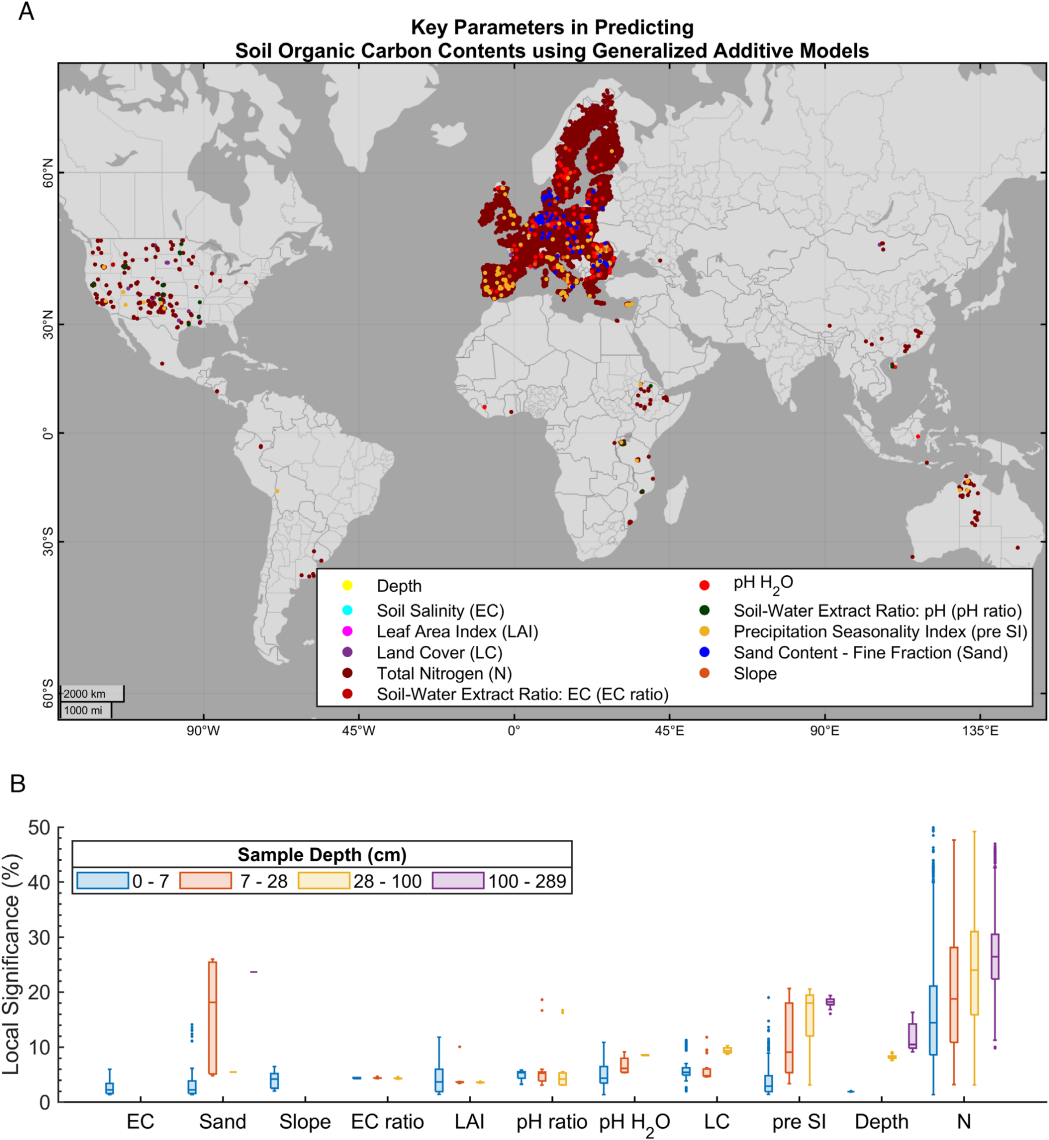
Covariates with the highest local significance in predicting SOC content using the GAMs. (*A*) the covariates with the highest significance in prediction of topsoil (0 to 7 cm) SOC content at each observation location. (*B*) box plot of the local significance of covariates at various depths. The boxes include the median, lower and upper quartiles, and nonoutlier minimum and maximum values. Outliers are calculated as values more than 1.5 times the IQR away from the top or bottom of the box. For every observation, the percentage of local covariate significance for predicting SOC was calculated by dividing the absolute effect of that specific covariate by the sum of the absolute effects of all other covariates, excluding the intercept.

The analysis of the most significant local covariates in prediction of SOC values revealed soil total N, land cover, and precipitation SI, and pH among main factors highly correlated with SOC content (Fig. 2). For a total of 30,216 observations, soil N emerged as the most significant covariate, with a mean importance of ~15.35% (SD = ~8.51%). Similarly to Evans, Burke, and Lauenroth (31), our study suggests that there is a strong correlation between SOC and N levels, given that N is a main component of soil OM (32). The results align with the findings of Xu et al. (33) or Doetterl et al. (34), who highlighted the significance of geochemistry as a key factor for soil C storage, in addition to climate. Similarly, for 1,660 soil samples, land cover exhibited a notable significance (mean importance of ~5.55%, SD = ~1.38%) followed by precipitation SI, for 645 soil samples (mean importance of ~3.82%, SD = ~2.52%), suggesting the primary factors explaining SOC variability are climate and land cover type. Previous studies have also identified soil moisture, clay content, or land cover as the primary covariates (32, 35–37).

## Relation between Soil Salinity and SOC

**Fig. 3.**
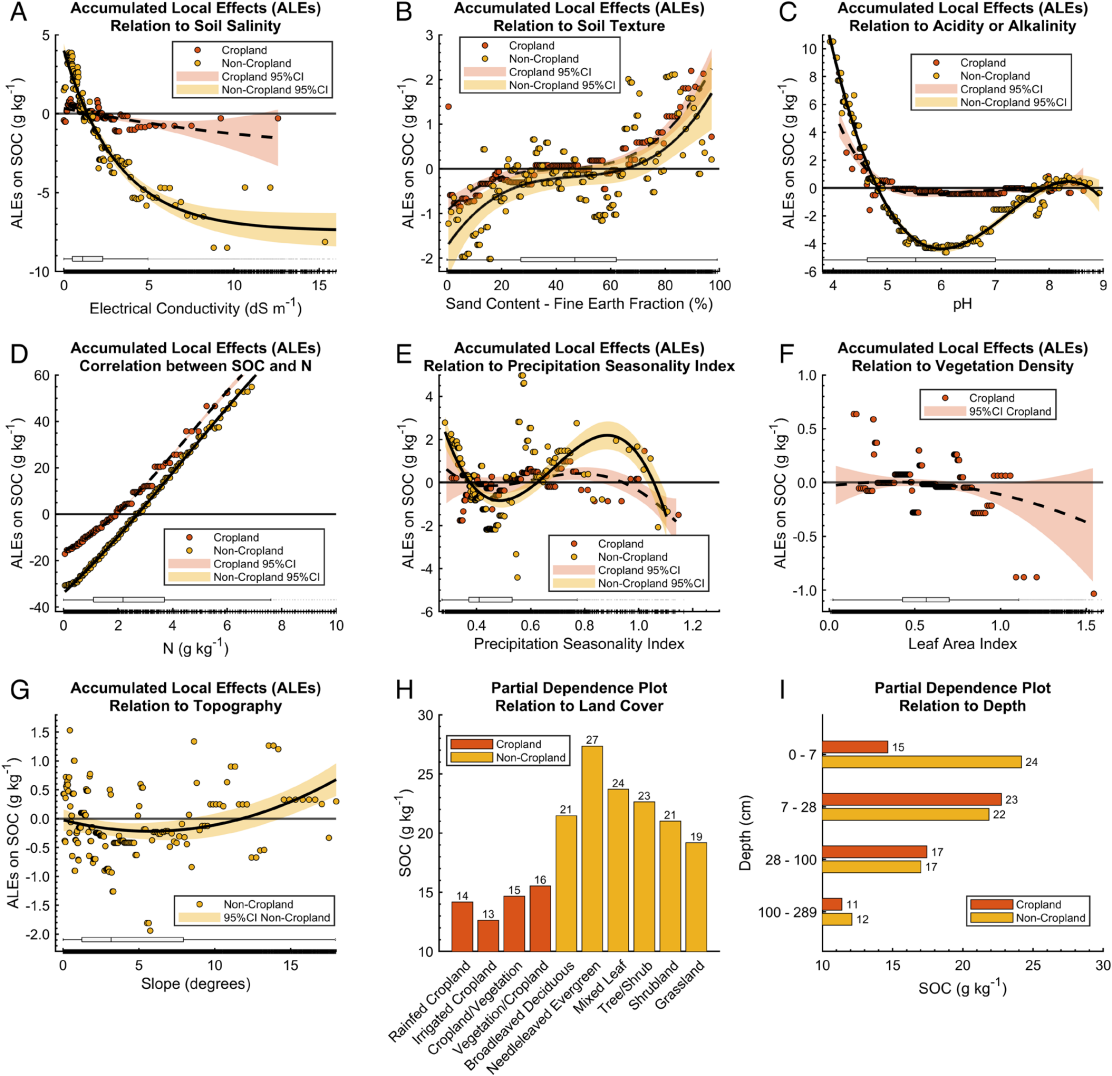
ALEs of covariates on predicted SOC content using the fitted GAMs. The *Y*-axis represents the deviation of GAMs’ predictions from the mean predicted SOC (18.47 and 35.96 g kg^−1^ for croplands and noncroplands, respectively) as the covariate varies from its lowest to highest values. Panels show the relation between SOC and (*A*) soil salinity, (*B*) soil sand content, (*C*) soil pH, (*D*) soil nitrogen content, (*E*) precipitation Seasonality Index, (*F*) Leaf Area Index, (*G*) terrain slope, (*H*) land cover type, and (*I*) soil depth, respectively. The ALE plots are computed by dividing the covariate feature into 200 sections. The covariates’ distribution is represented by the horizontal boxes close to the *x*-axis. The boxes represent the median, lower, and upper quartiles, and nonoutlier minimum and maximum values. Outliers are determined as values that exceed 1.5 times the interquartile range (IQR) away from either side of the box.

We used the developed GAMs to estimate the Accumulated Local Effects (ALEs) (38) of each covariate on the predicted SOC contents (Fig. 3). ALE plots allow understanding of how variation in each covariate influences the predicted outcome (SOC) while accounting for the interactions and nonlinearities with other covariates. We generated ALE plots for each covariate of interest. These plots display how the SOC deviates from the mean predicted SOC content as the covariate’s value ranges from its minimum to its maximum across the dataset.

Using ALEs, we found a general nonlinear decreasing trend in predicted SOC with an increase in soil salinity (Fig. 3*A*), for both crop and noncroplands. For croplands, ALEs are quantified by the equation ALE = 0.34 + −0.21EC + 0.01EC^2^ while for noncroplands, ALE = −7.42 + 11.43exp(−0.31EC). In those equations, ALE represents the deviation of SOC from the mean predicted SOC (18.47 and 35.96 g kg^−1^ for croplands and noncroplands, respectively), and EC is the soil salinity measured in dS m^−1^. This implies that higher soil salinity is associated with reduced SOC content, consistent with geographically specific investigations of Zhang et al. (39), Qu et al. (40), Setia et al. (41), and Zhao et al. (42). The equation also provides insight into the magnitude of the correlation between soil salinity and SOC content. For example, the equation for croplands suggests that a soil salinity increase from 1 to 5 dS m^−1^ is associated with a decrease in SOC from 0.14 g kg^−1^ above the mean predicted SOC (18.47 g kg^−1^) to 0.46 g kg^−1^ below the mean predicted SOC. Such increase in soil salinity is associated with a stronger decrease of 5.95 g kg^−1^ (ALE = 0.96 to ALE = −4.99) for noncroplands with mean SOC = 35.96 g kg^−1^. This finding confirms that soil salinity has dominantly a negative correlation with SOC content when controlling for the role of other covariates explaining the variability of SOC. It is important, however, to note that this is a correlative analysis. We use the spatial distribution/variability of both variables to see how they are correlated. Other factors, not included in their multivariate analysis, might affect the distributions of both variables (i.e., SOC and soil salinity).

The ALEs also reveal the relations between SOC and other relevant soil and environmental parameters. The source soil profile databases also have documented a nearly linear relationship between SOC and N (28) (Fig. 3*D*). For both crop and noncroplands. the partial dependence between SOC and sample depth shows a negative association between depth and SOC, aligning with findings from previous literature. These relationships not only validate the effectiveness of the GAM developed for our study but also shed light on other critical questions regarding interactions between carbon dynamics in the soil and the atmosphere.

High acidic soils (low pH) often have slower decomposition rates, leading to higher SOC content, while highly alkaline soils (high pH) can promote decomposition and reduce SOC accumulation (43, 44), which is to some extent reflected in the results presented in Fig. 3*C*. While some studies suggest a strong positive correlation between precipitation and SOC (31, 45, 46), others demonstrate the minimal to no impact that precipitation has on SOC (34, 47). The ALEs calculated here (Fig. 3 *E* and *F*) reveal that at low to moderate precipitation SIs, the relation between SOC precipitation SI is complicated, while in highly humid or high vegetation environments, characterized by elevated SI or LAI, the presence of abundant vegetation does not necessarily result in increased SOC content [e.g., Spain (48) or Lu, Gilliam, Guo, Hou, and Kuang (44)]. This counterintuitive phenomenon can be attributed to specific factors and processes influencing carbon dynamics in these ecosystems. Accelerated erosion, high decomposition rates, rapid leaching of OM, the presence of high-quality and easily decomposable litter, prolonged waterlogging, and anaerobic conditions contribute to an accelerated net loss of SOC despite the vegetation cover.

Our findings indicate that the role of soil texture in influencing SOC stocks is complex and varies with different fractions of the soil (Fig. 3*B*). Specifically, we observed that increase in soil sand content is positively correlated with SOC content, especially in the 0 to 20% and 60 to 100% ranges. Similar trends are observed in other studies such as Vos et al. (49) and Yang et al. (50). At low SOC levels, clay and silt particles predominantly hold the SOC. However, as SOC content increases, the proportion of SOC in the sand fraction also rises (50). This suggests that only soils with a high sand fraction have the ability to retain high levels of SOC. Diverse contrasting positive and negative correlations between soil texture and SOC content have been reported (47, 51–54). The range of these relationships highlights the significance of SOC composition (55, 56) and clay type (57) as critical factors in the interplay between SOC and soil texture, especially at lower SOC levels.

## Topsoil Organic Carbon Content Response to Increased Salinity

**Fig. 4.**
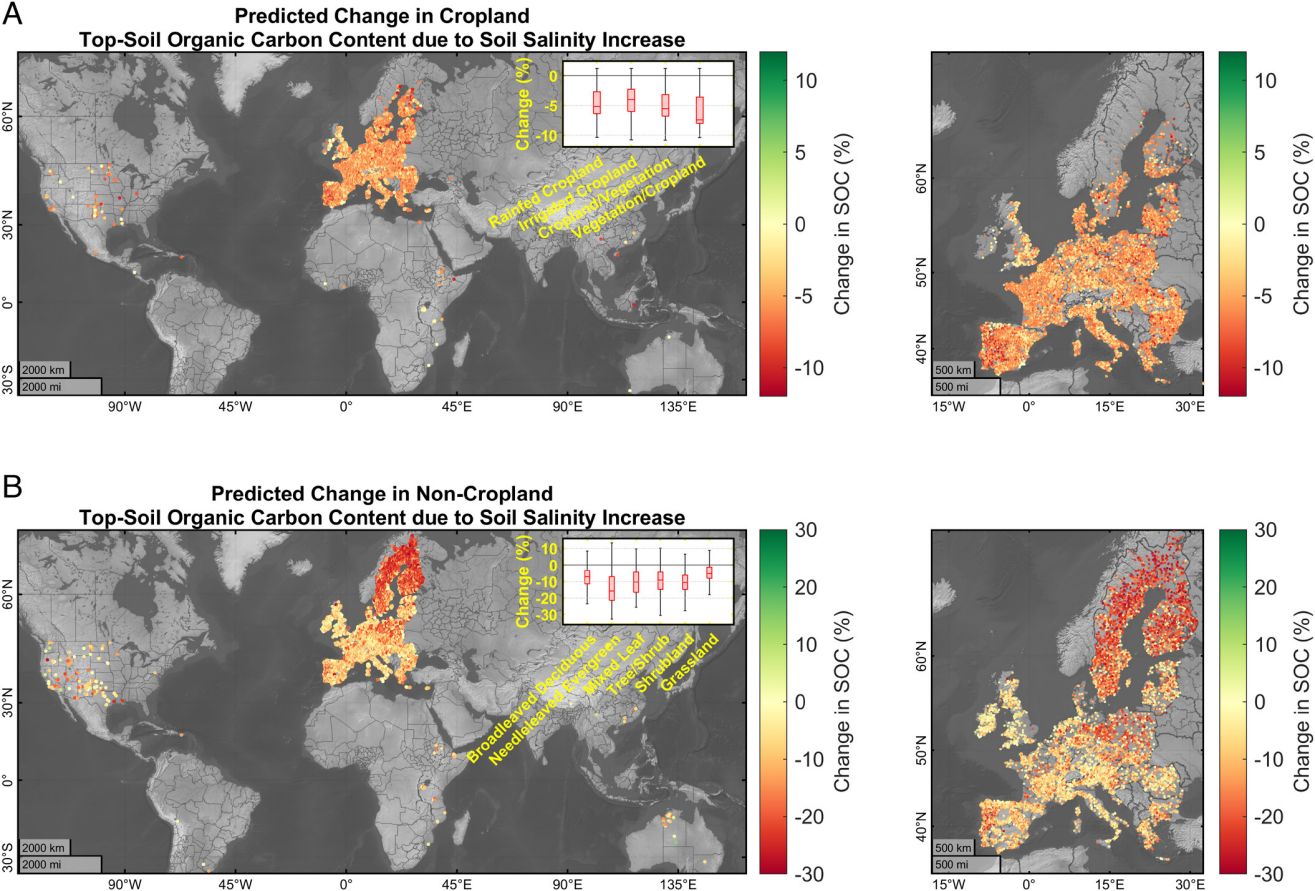
Impact of 1 SD increase in soil salinity on topsoil (0 to 7 cm) organic carbon (OC) content at the location of soil profiles/samples, (*A*) in croplands and (*B*) in non-croplands. The maps show the GAMs’ predicted increase in topsoil OC levels resulting from a 1 SD rise in soil salinity, indicating the sensitivity of topsoil OC to changes in salinity. The right-side panels show a higher zoom level in Europe, where the data derived from the LUCAS topsoil dataset are predominantly located. The box charts include the median, lower, and upper quartiles, and nonoutlier minimum and maximum change values for major land cover types. Outliers are calculated as values more than 1.5 times the interquartile range (IQR) away from the top or bottom of the box.

For dominant land cover types (shown on the *Insets*, Fig. 4), we employed the fitted GAMs to estimate the topsoil OC (0 ~ 7 cm) content in soil sample locations as a result of a 1 SD increase in soil salinity (Fig. 4). For croplands, the 1 SD increase in soil salinity corresponds to an approximately 263.2% increase in the median salinity (relative to original median = 4.32 dS m^−1^), while for noncroplands, it represents a nearly 245.24% increase in the median (original median = 3.83 dS m^−1^). The predictions, along with 95% CIs, were made both before and after the increase in salinity levels. Before the 1 SD increase in soil salinity, the 95% CIs for the predictions showed a median range of 12.48 and 22.78 g kg^−1^, for crop and noncroplands, respectively. However, after the increase in these parameters, the median 95% CIs were, respectively, 12.78 g kg^−1^ for soil salinity of croplands (*SI Appendix*, Fig. S2) and 21.6 g kg^−1^ for noncroplands (*SI Appendix*, Fig. S3). The results indicated higher uncertainty in the estimates for soil samples located in latitudes above 55°N, particularly in regions like Scandinavia and the northern United Kingdom.

For croplands, fitted GAM estimated a 1 SD increase in soil salinity is correlated with a decrease of approximately ~4.4% in the mean topsoil OC content of the soil samples (Fig. 4*A*), relative to the current mean predicted ${\overset{¯}{\mathrm{SOC}}}_{\mathrm{current}}$ of ~17.87 g kg^−1^. On the other hand, a 1 SD increase in soil salinity of samples located in noncroplands was estimated to be correlated with an increase of approximately ~9.26% in the mean topsoil OC content (Fig. 4*B*; ${\overset{¯}{\mathrm{SOC}}}_{\mathrm{current}}$ = ~36.01 g kg^−1^), in line with Setia et al. (13), who also estimated that world soils may lose 6.8 Pg of SOC by the year 2100 due to the projected increase in saline soils. To evaluate this, they used a modified Rothamsted Carbon model (58). Our results also indicated the variability in the relationship between soil salinity and SOC at different land covers. For croplands, the decrease in SOC in association with soil salinity was found to be little more noticeable (mean = ~−5.75%; ${\overset{¯}{\mathrm{SOC}}}_{\mathrm{current}}$ = ~23.71 g kg^−1^) in lands covered by a mosaic of natural vegetation and cropland (tree, shrub, herbaceous cover >50% while cropland <50%) and less in irrigated croplands (mean = ~−3.78%; ${\overset{¯}{\mathrm{SOC}}}_{\mathrm{current}}$ = ~13.16 g kg^−1^). However, for the noncroplands, the largest association was estimated for evergreen needle-leaved tree cover (mean = ~−14.25%; ${\overset{¯}{\mathrm{SOC}}}_{\mathrm{current}}$ = ~44.4 g kg^−1^) and the lowest was estimated for grasslands (mean = ~−4.58%; ${\overset{¯}{\mathrm{SOC}}}_{\mathrm{current}}$ = ~33.57 g kg^−1^).

In conclusion, this study provides insights into the complex interactions between soil salinity and SOC content across various land covers. The findings indicate that environmental factors that significantly explain SOC variability varying across different geographical regions and land use types. These results contribute to our understanding of the dynamics of SOC storage and have implications for land management strategies to mitigate the effects of changing environmental conditions on SOC levels. Analyzing the relationship between SOC content and soil salinity provides policymakers with insights to make informed decisions regarding sustainable land management, climate change mitigation, and agricultural practices. By integrating these findings into policies and management strategies, policymaking can use the results to promote soil health, enhance carbon sequestration, and contribute to global efforts to combat climate change.

## Methods

We began by gathering soil profile data from soil databases, including measurements of SOC and soil salinity. Additionally, we acquired a complete dataset of various environmental and soil physio-chemical properties that are known to correlate with the SOC pool over medium- to long-term periods.

Following the preparation of the dataset, we selected and used the most significant parameters as an input for developing GAMs. Using the GAMs, we could recognize the specific correlation between soil salinity and SOC content while accounting for the effects of other environmental and physio-chemical factors.

### Soil Profile Data.

We collected soil profile data, focusing on salinity measurements represented by EC (the ability of a soil–water extract to conduct electrical current: dS m^−1^) and total SOC content (g kg^−1^) in the fine earth fraction. The fine earth fraction refers to soil particles that are smaller than 2 mm in size. We obtained the data from two primary sources: the LUCAS 2015 (59) (https://esdac.jrc.ec.europa.eu/content/lucas2015-topsoil-data, accessed on 03-2024) and LUCAS 2018 (28, 32) (https://esdac.jrc.ec.europa.eu/content/lucas-2018-topsoil-data, accessed on 03-2024) topsoil survey data inventories, covering European Union countries, and the ISRIC—WoSIS Latest (dynamic) Standardized datasets (29, 60), providing global soil salinity measurements (https://www.isric.org/explore/wosis/accessing-wosis-derived-datasets, accessed on 03-2024).

The total SOC content in LUCAS inventories is measured using the ISO 11265:1994 Dry combustion method, while the soil salinity is determined using the ISO 11265:1994 method, which involves measuring the EC between metal electrodes in an aqueous extract of soil with a specific soil-to-water ratio (1:5 soil mass to water volume). On the other hand, the WoSIS dataset from ISRIC provides soil salinity measurements globally using different soil–water extract ratios: 1:2 (ELCO20), 1:2.5 (ELCO25), 1:5 (ELCO50), and saturated paste (ELCOSP) since 1920 to 2016.

### Parameters Correlated with SOC.

SOC content can be correlated to various geochemical and environmental parameters. These parameters can interact with each other and affect the dynamics of OC in the soil. We considered the following in our analysis:

#### Climate.

Factors such as temperature, precipitation, and evapotranspiration influence the activity of soil microorganisms responsible for OC decomposition (31, 45, 61, 62). To consider the role of precipitation, freezing days, and potential evapotranspiration on soil processes, we employed the CRU TS vs. 4.07 gridded monthly dataset at 0.5° spatial resolution (63). We used air temperature from the ERA5 monthly averaged data, providing air temperature records from 1940 to the present at 0.25° spatial resolution (64). The calculation of the precipitation SI for each year was based on Walsh and Lawler (65) method, using the following equation: $SI=\left(1/12\right)\sum_{n=1}^{n=12}\left|Pi-R/12\right|$, where *Pi* represents the monthly precipitation for month *i* (January to December). *R* is the total annual precipitation for the particular year under study, and *n* is the total number of months (12 mo in a year).

#### Nitrogen.

Since N is a main component of soil OM (32), the spatial-temporal distribution of N can closely correlate with that of OC. The total N content (g kg^−1^) of the soil samples was directly derived from three sources: LUCAS 2015, LUCAS 2018 topsoil survey data inventories (the total N includes ammonium-N, nitrate-N, nitrite-N, and organic N, measured using the ISO 11261:1995 method, modified Kjeldahl method), and the ISRIC—WoSIS Latest Standardized datasets (Kjeldahl method) (29, 66).

#### Vegetation.

The density of vegetation cover can significantly impact SOC content, especially through litter inputs (67, 68). High soil salinity can impact plant productivity and growth. However, we address vegetation cover as a distinct variable, averaged over the long term. The relationship between soil salinity and vegetation response is intricate, particularly at lower salinity levels (ELCOSP < 4 dS m^−1^). Furthermore, salinity is just one of the factors influencing vegetation. For example, other nutrients such as phosphorus and potassium availability also affect plant growth and development. We used LAI as a proxy to vegetation health and density. We used Version v3.0 10-daily gridded data from 1981 to the present (69), obtained from the Copernicus Climate Change Service, Climate Data Store (CDS). This dataset provided us with LAI at a horizontal resolution of 1/30° (~4 km, sensor: AVHRR) for the period between 1982 and 1998 and at a higher resolution of 1/112° (~1 km, sensor: VGT) for the years 1999 to 2020. Human activities such as tillage, crop rotation, application of fertilizers, and organic amendments can affect SOC content (70). We assumed that the relation between SOC and crop rotation, tillage system, nutrient availability, and fertilizer usage are reflected in parameters such as LAI and soil N.

#### Soil texture.

Soil texture refers to the soil’s relative proportions of sand, silt, and clay particles (53, 54). Soil texture affects factors like water-holding capacity, aeration, and microbial activity. The soil samples’ corresponding percentages of soil clay and sand in the fine earth fraction were obtained from LUCAS 2009 (71) (https://esdac.jrc.ec.europa.eu/content/lucas-2009-topsoil-data, accessed on 03-2024) and LUCAS 2015 topsoil survey data inventories, as well as the ISRIC—WoSIS Latest Standardized datasets (29). Due to the absence of soil texture data in the LUCAS 2018 inventory, we used the samples and locations from the 2015 campaign. This means we excluded locations sampled during the LUCAS 2018 campaign that were not sampled in 2015.

#### Soil pH.

Soil pH influences the activity of soil microorganisms involved in OM decomposition. The pH in H_2_O of the soil samples was obtained from three sources: LUCAS 2015, LUCAS 2018 topsoil survey data inventories (ISO 10390:2005; glass electrode in a 1:5 [V V^−1^] suspension of soil in H_2_0), and the ISRIC—WoSIS Latest Standardized datasets (29). WoSIS includes the soil-to-water ratio of the pH measurement solution as part of its metadata.

#### Drainage and soil moisture.

The drainage characteristics and soil moisture regime can impact SOC content (36). Poorly drained soils with waterlogging conditions limit oxygen availability, leading to slower decomposition rates and higher SOC content (72). Conversely, well-drained soils may have higher decomposition rates, potentially reducing OC levels. As representative indicators of the volume of water and soil moisture at varying soil depths (V V^−1^), we used ERA5 monthly averaged data on single levels from 1940 to present (64) volumetric soil water at layers 1 (soil depth 0 to 7 cm), 2 (7 to 28), 3 (28 to 100), and 4 (100 to 289) obtained from Copernicus Climate Change Service (C3S) CDS. Due to the challenges in acquiring reliable data on soil drainage properties, soil texture, and moisture data were assumed as a proxy for soil drainage capacity.

#### Topography.

It influences various environmental factors that affect SOC dynamics, such as soil moisture, temperature, nutrient availability, and soil erosion rate (73). To account for the role of topography, we obtained topographic variables, including elevation and slope, at the location of each soil sample/profile. We used the MERIT DEM (https://hydro.iis.u-tokyo.ac.jp/~yamadai/MERIT_DEM/, accessed on 03-2024)—Multi-Error-Removed Improved-Terrain Digital Elevation Model—offering elevation data at a resolution of 3 arc s (~90 m at the equator) (74). The topographic data were processed and analyzed within the Google Earth Engine environment (75).

#### Land cover.

Land cover changes, such as deforestation or conversion of natural ecosystems to agriculture, can lead to the loss of OC from the soil (70, 76). We used yearly land cover classification gridded maps from 1992 to the present (versions 2.0.7cds and 2.1.1) derived from satellite observations provided by Copernicus Climate Change Service (C3S) CDS (77) at 300 m resolution to identify the land cover dynamic globally. The dataset offers global land surface maps with 22 classes, defined using the United Nations Food and Agriculture Organization’s Land Cover Classification System (LCCS). Due to data unavailability for land cover information before 1992, we limited our analysis to soil observations collected after 1992. Samples from the first four classes, 10, 20, 30, and 40 of the C3S LCCS were considered cropland observations, while the rest were categorized as noncroplands.

### Statistical Analysis—GAM Fitting.

The parameters explained above underwent initial data screening, which involved checking the plausible range of values (*SI Appendix*, Fig. S4). Soil samples from WoSIS provided information on the upper and lower sampling depths. We calculated the mean of these depths and grouped the samples into four layers, following a similar approach to ERA5 reanalysis (64) soil layers—see “*Drainage and Soil Moisture*” in Parameters Correlated with SOC. The soil volume of water data was then associated with each sample based on these layers. Although the LUCAS soil samples represented the physio-chemical properties of the top 0 to 20 cm and their mean depth (10 cm) fell within group 2 (7 to 28 cm), we assigned all LUCAS data to group 1 (0 to 7 cm) for consistency and to align with the data’s characteristic representation of this specific depth range.

In essence, our assumption was that a steady-state balance exists between soil, land, and climate at the sampling locations, and our objective was to evaluate the variability of SOC in relation to soil salinity. Organic soil samples with a SOC above 150 were excluded (*n* = 2,050). Specific parameters like climate-related factors and vegetation exhibit higher dynamism over time and may not directly relate to the SOC content at the time of sample acquisition. To address this and reduce noise in the data, we used the long-term average of these parameters on SOC content. We aggregated dynamic covariates to annual means or accumulations over a window size of 10 y (left side, i.e., 10 y before soil sampling date) and then associated these values with the corresponding soil samples.

To analyze the relationship between soil salinity and SOC, we fitted two GAMs (78), one for observations located in croplands and one for the ones in noncroplands. We employed the MATLAB (79) “fitrgam” function (https://uk.mathworks.com/help/stats/fitrgam.html, accessed on 03-2024) for fitting the models. We favored GAM over more interpretable models like generalized mixed linear models to capture the nonlinear interactions between the terms and the variable (SOC) and to handle the missing values. In a GAM, the relationship between the independent variable and predictors can be nonlinear. This is achieved by introducing smooth (shape) functions of the predictor terms into a linear model (80). Important interaction terms can be represented by bivariate shape functions, allowing the incorporation of interactions between covariates (81). We avoided using more complex and flexible models like boosted ensembles of regression trees or Neural Networks to ensure the model’s interpretability. GAM models provide local effects of each term, offering insights beyond global predictor importance.

The GAMs assume an additive structure, requiring uncorrelated covariates in a nonlinear sense. Concurvity, similar to multicollinearity in linear models, introduces instability in GAM parameter estimates (82, 83). There might be innate correlations between selected covariates at large scale and the possibility of concurvity, e.g., increasing temperatures will impact vegetation, precipitation, and land management, all of which will feed back to SOC. To address model complexity and identify important covariates while minimizing redundancy, we applied the MRMR algorithm (30) using the “fsrmrmr” function in MATLAB (https://uk.mathworks.com/help/stats/fsrmrmr.html, accessed on 03-2024). This method, based on the Hilbert–Schmidt independence criterion, aims to address the issue of concurvity using the mutual information of the target variable and covariates and select a set of covariates that correlate well with the target variable yet remain uncorrelated with each other (84). Low-ranked covariates were removed from the analysis if the coefficient of determination (*R*^2^) for the fitted models, including or excluding those covariates remained unchanged. Moreover, we assigned weight to covariates that ranked high in both croplands and noncroplands to minimize the likelihood of chance findings. The results of covariate selection are presented in *SI Appendix*, Fig. S1.

To ensure data reliability, samples with over two missing covariates were excluded (*SI Appendix*, Fig. S5). During the fitting of the GAMS, we treated the soil-to-water ratio for measuring pH and the soil–water extract ratio for measuring soil EC as categorical variables, representing the methods used for pH and salinity measurements. We considered land cover as an additional categorical variable. The observations were given equal weight, and the response variable (SOC) was log10 transformed to address the right-skewness in the data. We included the interaction terms in fitted GAMs. While fitting the GAMs to the training sets, we optimized the hyperparameters using MATLAB’s built-in Bayesian optimizer with the “expected-improvement-per-second-plus” acquisition function. We employed a hold-out cross-validation scheme, with 25% of the data being held out for each evaluation. The objective function evaluation was repeated 30 times to ensure the robustness and reliability of the results.

The results of the 10-fold cross-validation for the final GAMs are presented in *SI Appendix*, Figs. S6–S9. For model in croplands: *R*^2^ = 0.83, RMSE = 6.57 g kg^−1^, Mean Absolute Error = 3.29 g kg^−1^, and Mean Bias = −0.63 g kg^−1^ and for model in noncroplands: *R*^2^ = 0.87, RMSE = 11.23 g kg^−1^, Mean Absolute Error = 6.56 g kg^−1^, and Mean Bias = −1.08 g kg^−1^. Estimating the effects of the covariates in predicting SOC when interactions are present can be challenging and complex. Therefore, for Figs. 1 and 2, which show the local significance of the covariates in predicted SOC values, we fitted separate GAMs excluding interaction terms. However, for the calculations of ALEs (38) and predictions of SOC resulting from changes in soil salinity, as shown in Figs. 3 and 4, we used the GAMs with interaction terms. These models demonstrated higher accuracy in predicting SOC compared to the model without interactions. Note that the predictions were exclusively made for the soil samples with complete data of all covariates. In our analysis, for continuous variables, we opted to use ALE plots for assessing the role of covariates on the predicted SOC content. In datasets with interrelated or correlated covariates, ALEs are preferred to PDPs (partial dependency plots).

### Study Limitations: Constraints and Considerations.

#### Concurvity.

The MRMR algorithm, employed for covariate selection, focuses solely on assessing pairwise independence among features. Consequently, it cannot address scenarios where one feature’s accurate estimation relies on a combination of several other features. In essence, these algorithms do not account for multivariate concurvity (84).

#### Different native spatial resolutions.

The difference in native spatial resolution of the covariates used in GAMs could introduce uncertainties and limitations in the interpretation of results. Covariates with coarser resolutions, such as climate data at the 0.5° spatial resolution, may capture regional or larger-scale climatic patterns but might not fully represent the microclimatic conditions directly influencing the soil sample location. Similarly, LAI data at a 4 km resolution may provide an average representation of vegetation cover within a larger area, potentially overlooking finer-scale variations in vegetation density that could affect SOC content. In addition to the differences in spatial resolution, the coarse resolution of covariates such as temperature and precipitation may be a potential reason why they don’t emerge as important covariates in the MRMR algorithm, although precipitation SI (0.5°) was ranked as an important covariate for both crop and noncroplands. It is possible that many samples are labeled with the same air temperature and/or precipitation values, leading the algorithm to assume that these parameters cannot sufficiently explain the variability in SOC. This limitation necessitates the need for finer-resolution data. It would be intriguing to use land cover information gathered in LUCAS soil surveys as a potential substitute for the C3S LCCS data to remove the innate uncertainties of satellite products.

#### Simplified representation of vegetation.

The vegetation in the study was primarily represented by LAI and land cover which serve as proxies for vegetation density. However, the specific vegetation types and the litter input to the soil can also influence SOC dynamics (68, 85).

#### Geographical bias.

Despite efforts to gather a global dataset representative of various land covers and biomes, most soil samples/profiles are located in Europe and originate from the LUCAS soil database. This geographical bias may limit the generalizability of the findings to other regions with different environmental conditions and management practices.

#### Limited control for soil aggregation.

The study controlled for the role of soil texture, especially sand content, on SOC variation. Still, it did not account for soil aggregation—the clumping of soil particles into larger structures or aggregates. The content of coarse fragments (>2 mm) can serve as a proxy for soil aggregation tendency, which was not included in the analysis due to data unavailability.

#### Experimental errors in SOC measurements.

Variation in SOC measurements may be attributed to systematic experimental errors related to the different methods used for measuring SOC content, such as the Walkley-Black Method, Dry Combustion, and Loss-on-Ignition. Similar limitations and advantages exist for other relevant covariates, particularly soil N content (e.g., Kjeldahl and modified Kjeldahl methods). More standardized and comprehensive measurement protocols would enhance the accuracy and comparability of SOC data.

#### Causal analysis.

Our analysis provides insights into the correlative relationships between soil salinity and SOC variability; however, further research is warranted to explore causality and address considerations related to the timescale. For instance, as soil salinity increases, plant communities may shift toward species more tolerant to salinity, keeping litter input into the soil. Similarly, microorganisms can adapt to long-term high salt exposure, undergoing genetic and physiological changes to cope with salinity stress, thereby maintaining metabolic activities and SOM mineralization (12, 18). This may involve longitudinal field experiments or controlled laboratory studies that manipulate soil salinity levels and monitor SOC dynamics over extended periods.

## Supplementary Material

Appendix 01 (PDF)

## Data Availability

The codes used in this study were developed using the MATLAB programming interface and are available at https://doi.org/10.6084/m9.figshare.23868531 (86). Previously published data were used for this work and appropriate acknowledgments and citations for the original sources are provided in the “*Parameters Correlated with SOC*” section of the *Methods* section. The final input into the GAM, including the averaged values of covariates at the soil profile/sample locations over a 10-y window can be accessed at https://doi.org/10.6084/m9.figshare.23868531 (86).
