# Supplementary material for: Negative correlation between soil salinity and soil organic carbon variability
Source: Proc Natl Acad Sci U S A. 2024 Apr 26;121(18):e2317332121. doi: 10.1073/pnas.2317332121 (PMC11067061; doi:10.1073/pnas.2317332121)
Supplement: Supplementary file 1 — Appendix 01 (PDF) [file pnas.2317332121.sapp.pdf]

Supplementary Information Appendix

Negative Correlation between Soil Salinity and Soil Organic Carbon Variability

Amirhossein Hassani[1], Pete Smith[2], Nima Shokri[3]

1. The Climate and Environmental Research Institute NILU, P.O. Box 100, Kjeller 2027, Norway
2. Institute of Biological and Environmental Sciences, School of Biological Sciences, University of Aberdeen, 23 St Macher Drive, Aberdeen, AB24 3UU, UK
3. Institute of Geo-Hydro-informatics, Hamburg University of Technology, Am Schwarzenberg-Campus 3 (E), 21073 Hamburg, Germany

*Correspondence: ahas@nilu.no; nima.shokri@tuhh.de

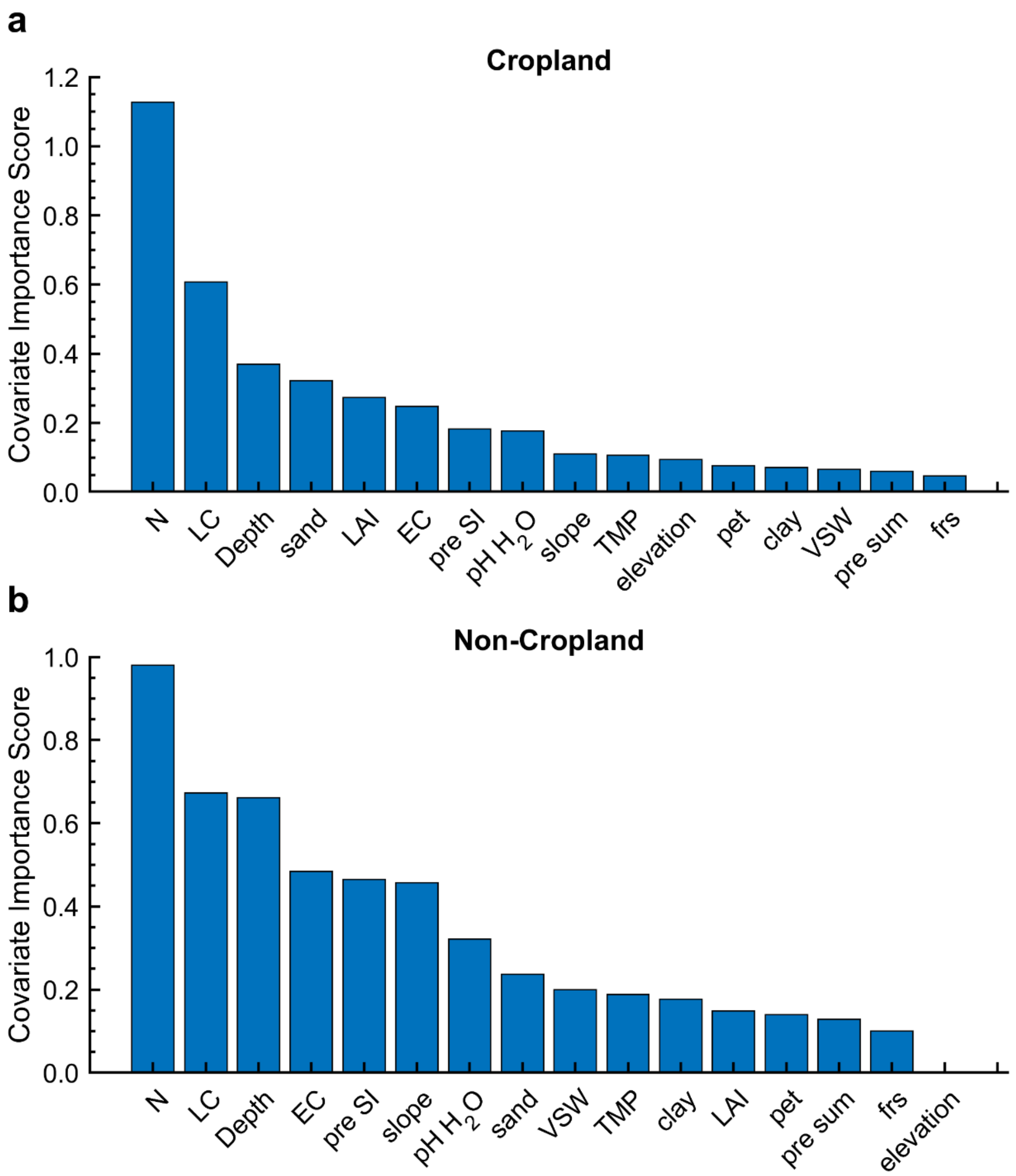

***SI Appendix*, Fig. S1: Most significant covariates for regression identified by Minimum Redundancy Maximum Relevance (MRMR) algorithm.** The drop in score between the first and second most important covariates is large, while the drops after the eighth covariates are relatively small. A large score value indicates that the corresponding covariate is important. Also, a drop in the covariate importance score represents the confidence of covariate selection. For example, if the software is confident of selecting a covariate x, then the score value of the next most important covariate is much smaller than the score value of x. The small drops indicate that the differences in covariate importance are not significant. The full definition of the covariates on x-axis are described in *SI Appendix*, Fig. S4. The first eight important covariates were selected based on their significance in predicting SOC content and minimal redundancy in covariate set.

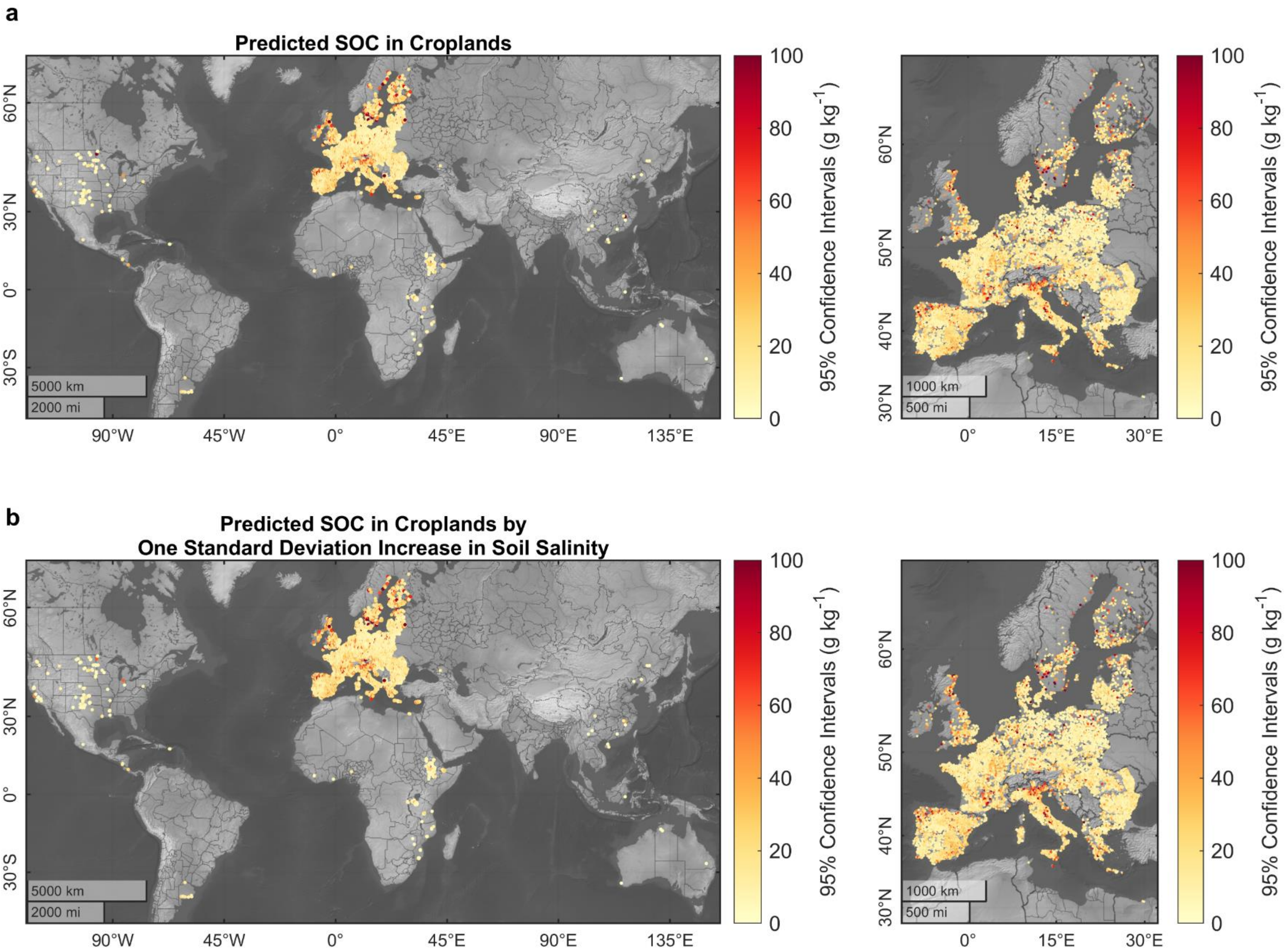

***SI Appendix*, Fig. S2: Range of 95% Confidence Intervals for predicted topsoil (0 - 7 cm) Organic Carbon (OC) content in croplands while keeping other covariates constant. a**, the predicted SOC for the current soil salinity levels at each point; **b**, the predicted SOC when salinity is increased by one standard deviation at the location of each point. The panels on the right provide zoomed-in maps of the Europe region for better visualization.

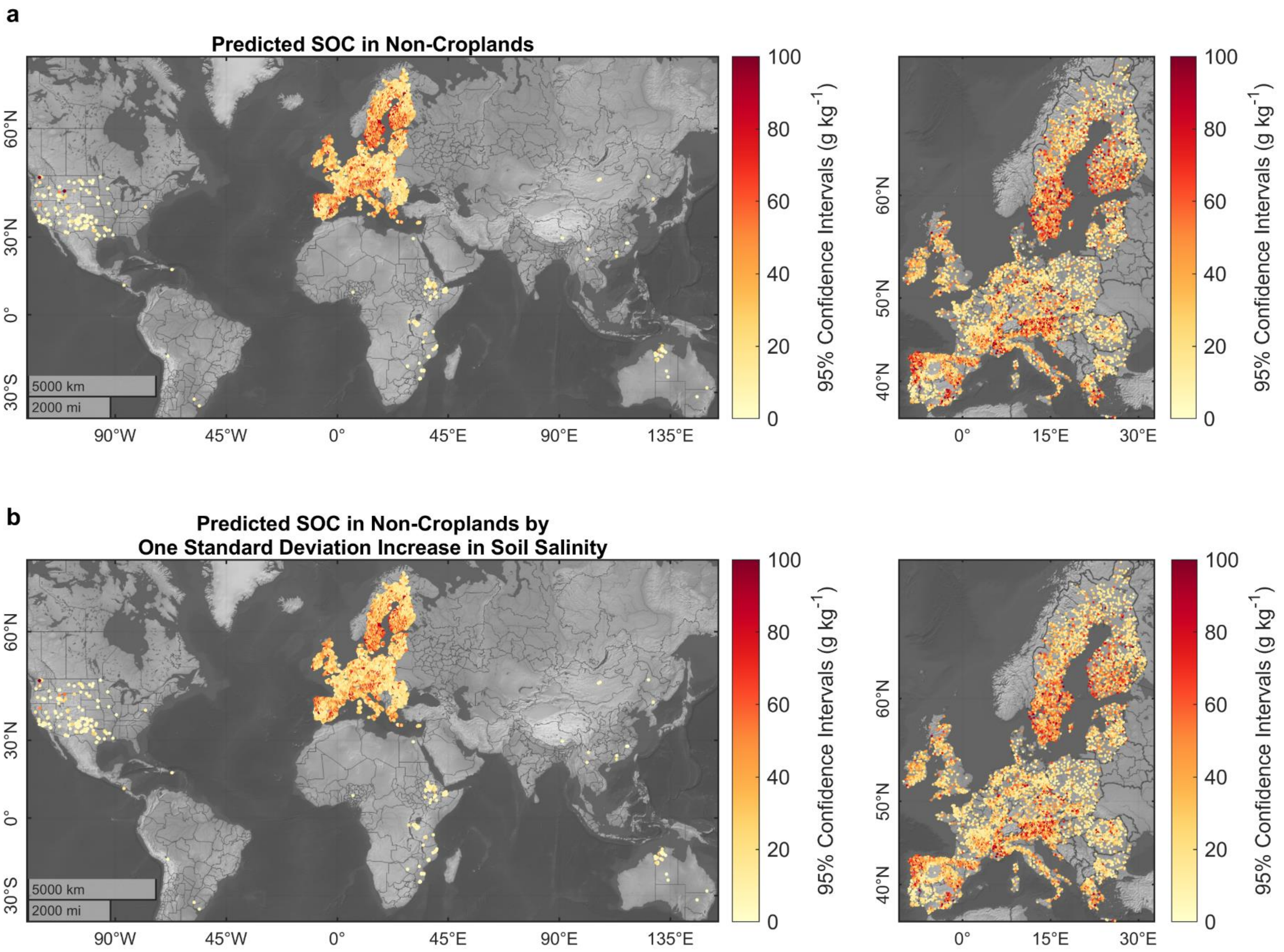

***SI Appendix*, Fig. S3: Range of 95% Confidence Intervals for predicted topsoil (0 - 7 cm) Organic Carbon (SOC) content in non-croplands while keeping other covariates constant. a**, the predicted SOC for the current soil salinity levels at each point; **b**, the predicted SOC when the air temperature is increased by one standard deviation at the location of each point. The panels on the right provide zoomed-in maps of the Europe region for better visualization.

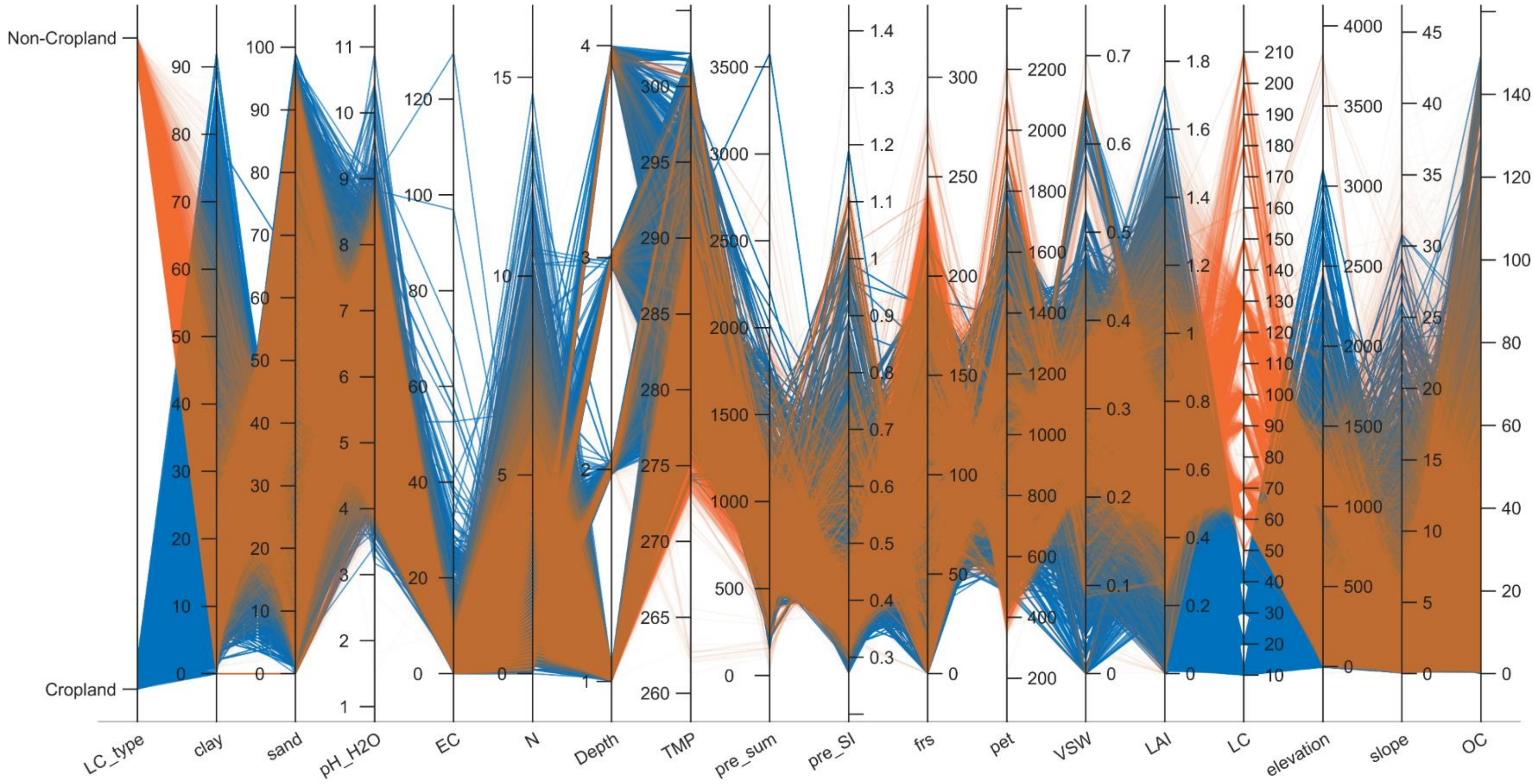

***SI Appendix*, Fig. S4: Range of covariates used to analyze the relation between soil salinity and Soil Organic Carbon (SOC).** The units of the variables from the left to right are: Main land cover types: classes, clay content in fine earth fraction: %, sand content in fine earth fraction: %, soil pH in H_2O: unitless, Electrical Conductivity: dS m^{-1}, soil total nitrogen (N) content: g kg^{-1}, depth category: 4 categories, annual daily mean air temperature: °K, annual rainfall: mm yr^{-1}, annual precipitation Seasonality Index: unitless, total number of freezing days per year: days, annual potential evapotranspiration: mm yr^{-1}, volumetric soil water content: unitless (V V^{-1}), Leaf Area Index: unitless, land cover types within the main land cover types: classes, elevation: meter, slope: degree, Soil Organic Carbon content: g kg^{-1}.

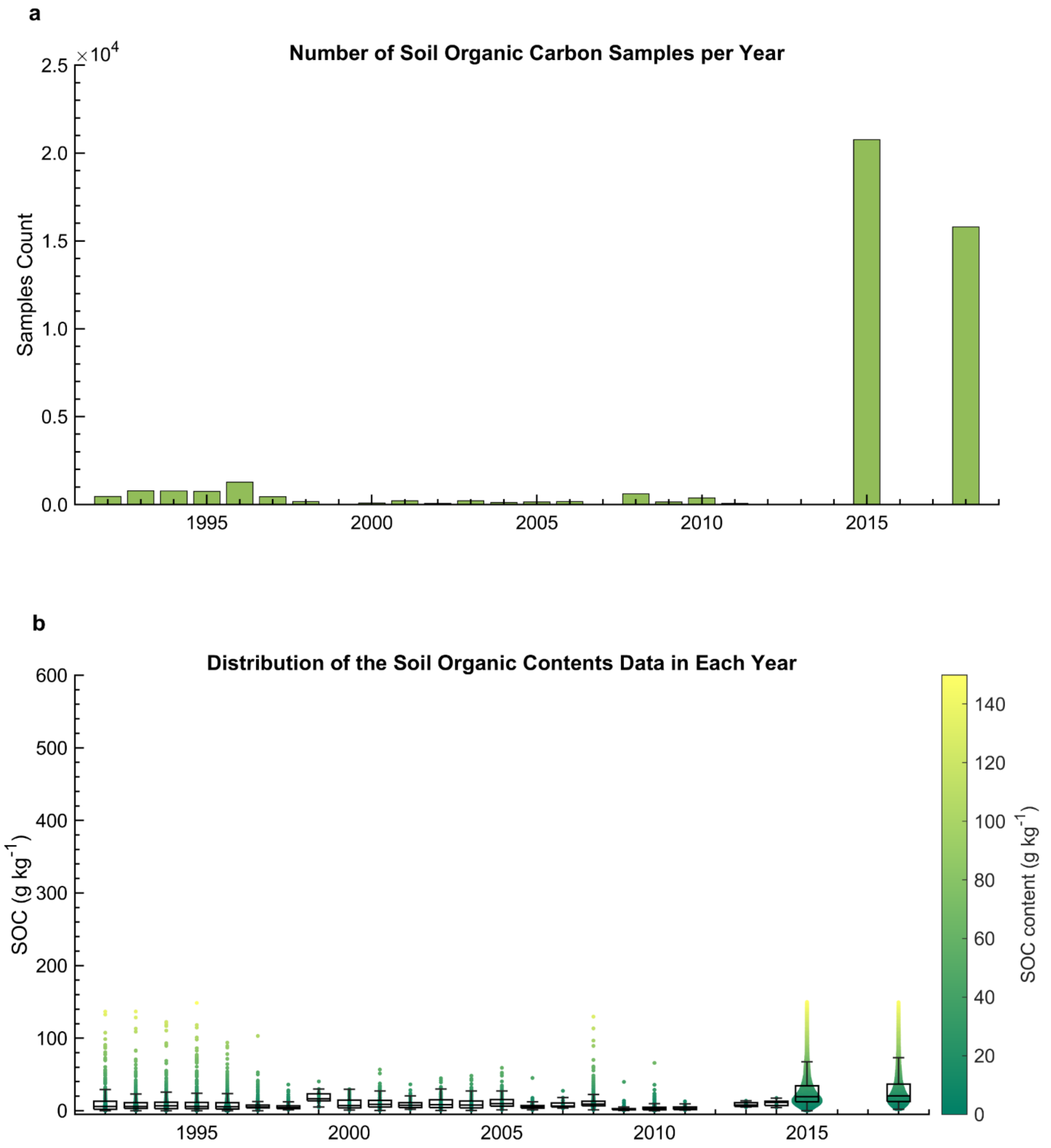

***SI Appendix*, Fig. S5: Distribution and number of Soil Organic Carbon (SOC) samples per year in the final dataset used for fitting the General Additive Models. a**, each bar corresponds to a specific year, and its height represents the number of SOC samples available for analysis. **b**, the boxes include the median, lower and upper quartiles, and non-outlier minimum and maximum values. Outliers are calculated as values more than 1.5 times the IQR away from the top or bottom of the box.

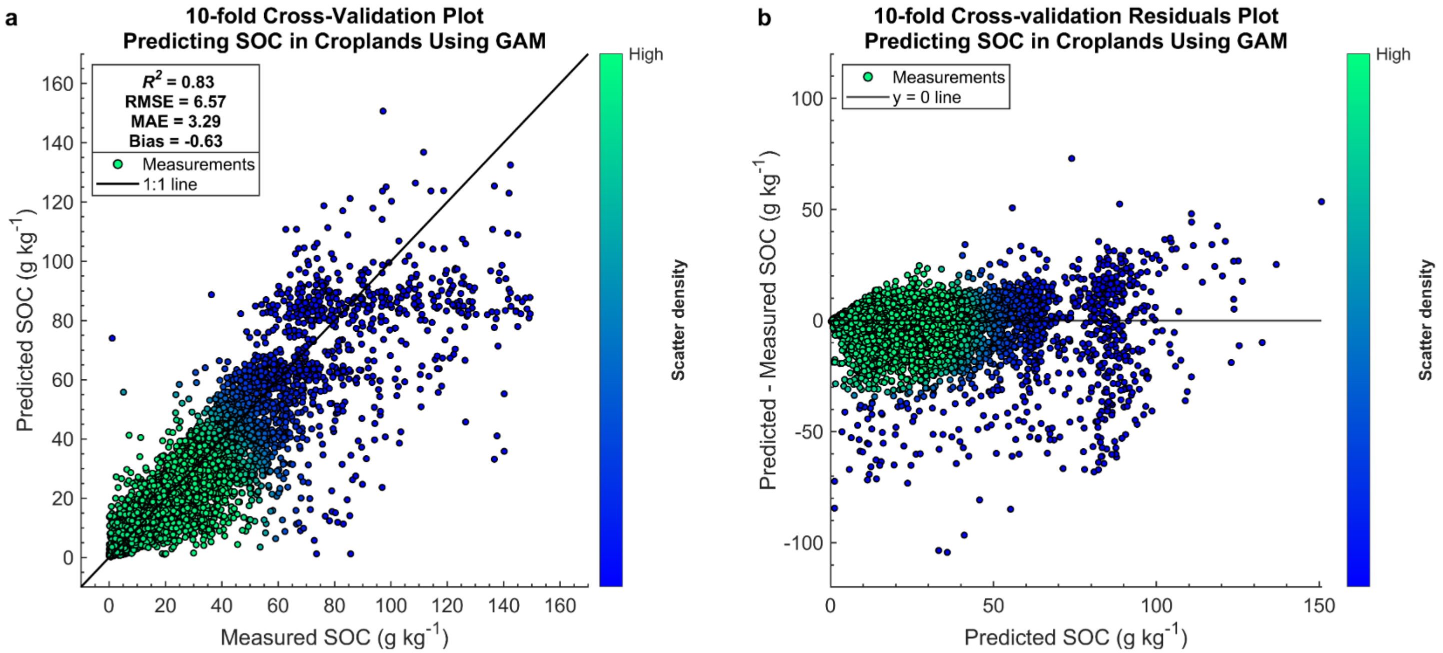

***SI Appendix*, Fig. S6: Results of 10-fold cross-validation for the final General Additive Model (GAM) fitted to the input data originated from croplands. a**, the validation plot with predicted SOC values plotted against measured SOC values, indicating the model's predictive performance. **b**, the residuals vs. the predicted SOC using the GAM, helping assess the model's accuracy and any error patterns. A considerable number of soil samples exhibit similar or very close values of SOC. As a result, the residual plots, which show the difference between the predicted and measured SOC values, display a linear pattern when the predictions are extremely low or high. Note that all calculations, including model cross-validation and residual analysis, were carried out after back-transforming the response variable (SOC) from logarithmic space. The logarithmic transformation was applied to address the original data's right-skewness and improve the model's performance.

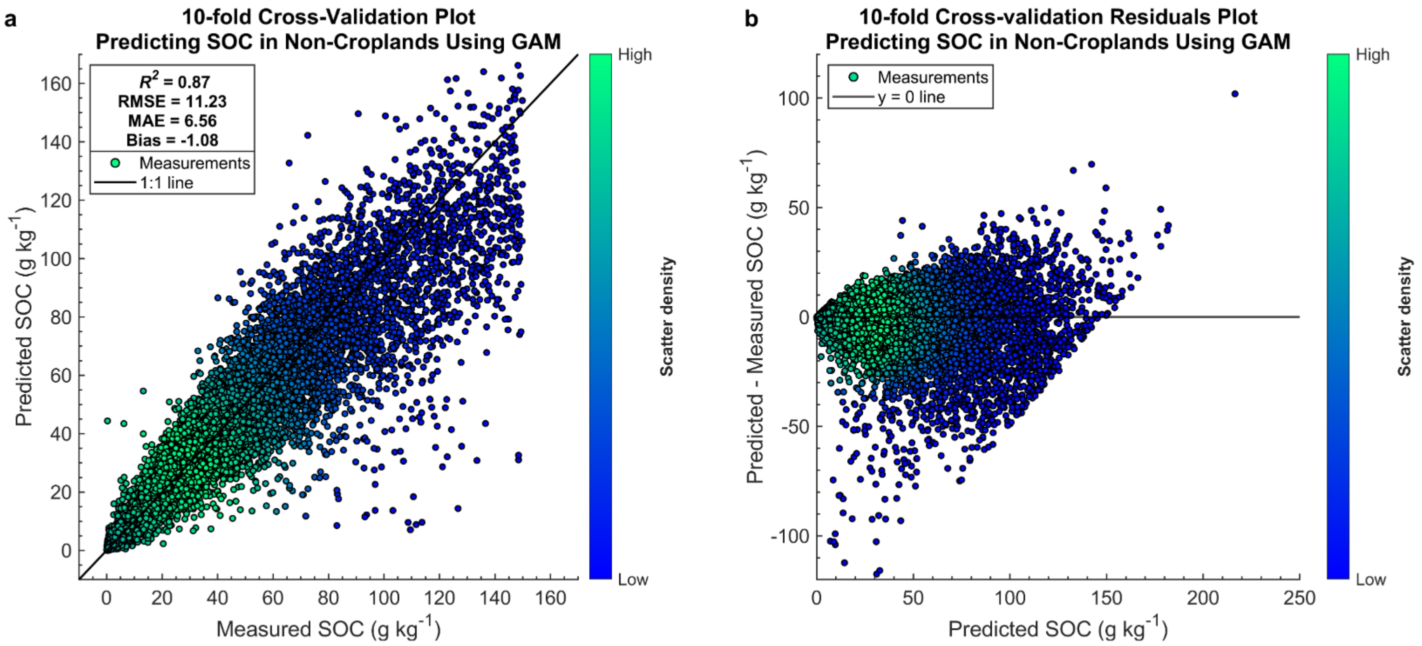

***SI Appendix*, Fig. S7: Results of 10-fold cross-validation for the final General Additive Model (GAM) fitted to the input data originated from non-croplands. a**, the validation plot with predicted SOC values plotted against measured SOC values, indicating the model's predictive performance. **b**, the residuals vs. the predicted SOC using the GAM, helping assess the model's accuracy and any error patterns. A considerable number of soil samples exhibit similar or very close values of SOC. As a result, the residual plots, which show the difference between the predicted and measured SOC values, display a linear pattern when the predictions are extremely low or high. Note that all calculations, including model cross-validation and residual analysis, were carried out after back-transforming the response variable (SOC) from logarithmic space. The logarithmic transformation was applied to address the original data's right-skewness and improve the model's performance.

a

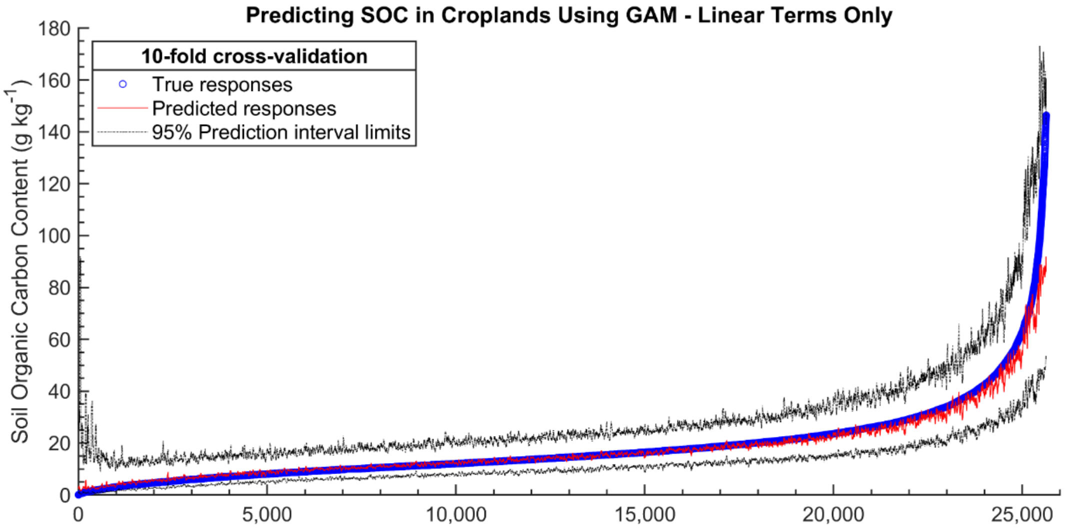

b

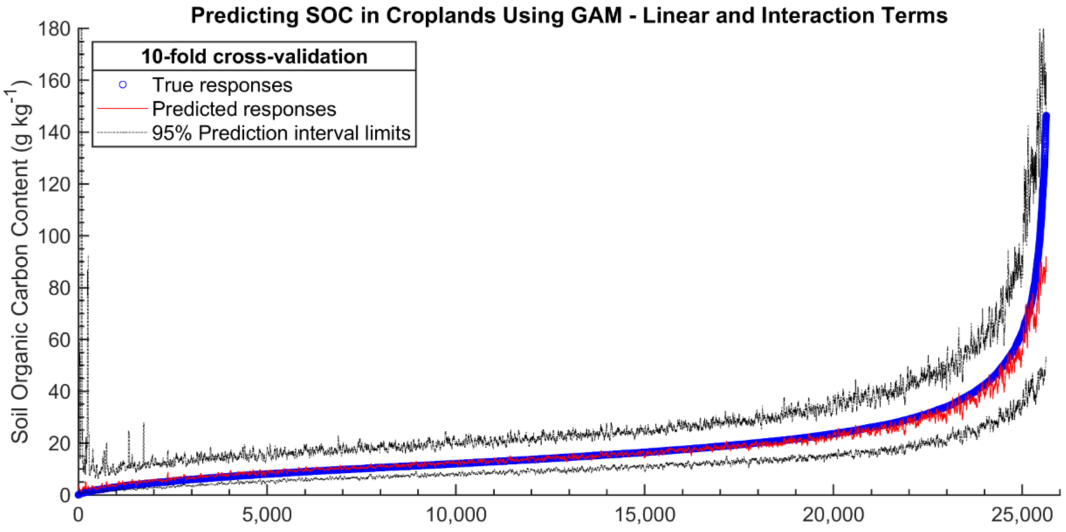

***SI Appendix*, Fig. S8: 10-Fold Cross-Validation of Soil Organic Carbon (SOC) contents predicted by the cropland model, with and without Interaction Terms.** The figure illustrates the results of 10-fold cross-validation for the predictions of SOC using the final fitted General Additive Model (GAM). The samples are sorted based on the magnitude of SOC values, and the x-axis represents the number of samples. In panel **b**, the predictions are made by including interaction terms in the GAM model.

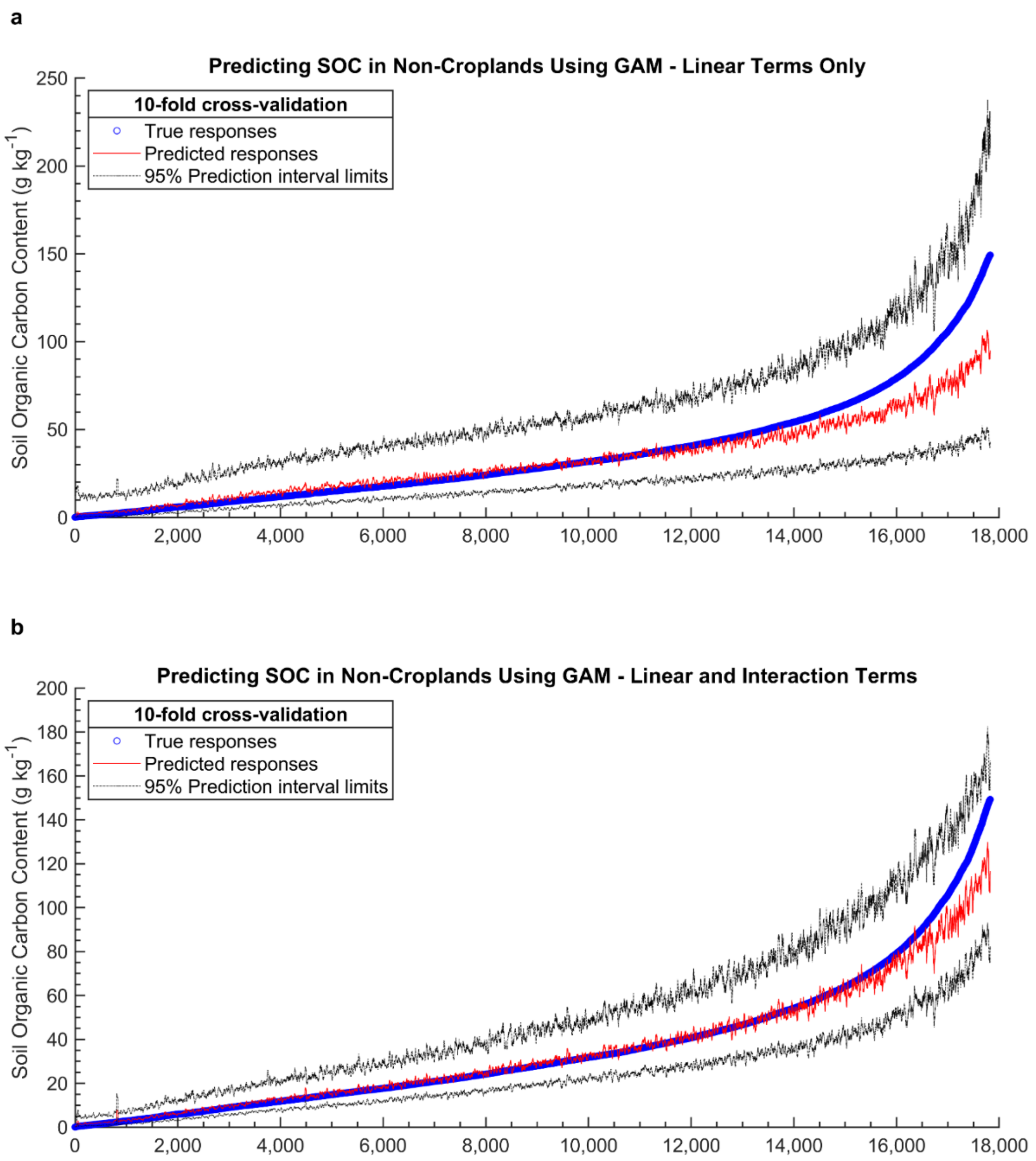

***SI Appendix*, Fig. S9: 10-Fold Cross-Validation of Soil Organic Carbon (SOC) predicted by the non-cropland model, with and without Interaction Terms.** The figure illustrates the results of 10-fold cross-validation for the predictions of SOC using the final fitted General Additive Model (GAM). The samples are sorted based on the magnitude of SOC values, and the x-axis represents the number of samples. In panel **b**, the predictions are made by including interaction terms in the GAM model. It is evident that the model with interaction terms provides higher accuracy in predicting SOC values compared to the model without interactions, especially for large SOC values.
